# Association of stress, depression, and suicidal ideation with subjective oral health status and oral functions in Korean adults aged 35 years or more

**DOI:** 10.1186/s12903-017-0391-4

**Published:** 2017-06-23

**Authors:** Young Sun Kim, Han-Na Kim, Jung-Ha Lee, Se-Yeon Kim, Eun-Joo Jun, Jin-Bom Kim

**Affiliations:** 1 Department of Nursing, College of Nursing, Catholic University of Pusan, 57, Oryundae-dae, Geumjeong-gu, Busan, 46252 Republic of Korea; 2 Department of Dental Hygiene, College of Health Sciences, Cheongju University, 298, Daesung-ro, Cheongwon-gu, Cheongju, 28503 Republic of Korea; 3 Department of Preventive and Community Dentistry, School of Dentistry, Pusan National University, 49 Busandaehak-ro, Mulgeum-eup, Yangsan City, Gyeongsangnam-do 50612 Republic of Korea; 4 BK21 PLUS Project, School of Dentistry, Pusan National University, 49 Busandaehak-ro, Mulgeum-eup, Yangsan City, Gyeongsangnam-do 50612 Republic of Korea

**Keywords:** Depression, Oral function, Stress, Subjective oral health status, Suicidal ideation

## Abstract

**Background:**

Oral health greatly affects well-being throughout the different stages of life from childhood to late adulthood. Loss of teeth due to poor oral health hinders mastication, leading to poor nutrition absorption, and affects pronunciation and aesthetics, leading to interpersonal difficulties. As social activities become limited, a sense of isolation and loneliness, stress, and depression grows while happiness decreases. This study aimed to examine the association of stress, depression, and suicidal ideation with oral health status and oral functions in a large nationwide sample of Korean adults aged 35 years or more.

**Methods:**

The sample comprised 15,716 adults, selected using a rolling survey sampling method and data were extracted from the Fifth Korea National Health and Nutrition Examination Survey (KNHANES) (2010–2012). Participants were interviewed about their self-evaluation of health including oral health status and mental health, such as stress, depression, and suicidal ideation. Data from 11,347 adults were finally selected after excluding participants with missing answers. The dependent variables were stress, depression, and suicidal ideation. The independent variables were gender, age, household income, education, smoking, drinking, oral health perception, chewing, and speaking. Complex samples logistic regression analyses were used to estimate odds ratios (ORs) and 95% confidence intervals (CIs).

**Results:**

Participants met the criteria for stress (25.4%), depression (13.0%), and suicidal ideation (13.9%). Subjective oral health status was not significantly associated with stress, depression, and suicidal ideation. However, the presence of very uncomfortable chewing problems was significantly associated with stress (OR = 2.294, 95% CI = 1.41, 3.72), depression (OR = 3.232, 95% CI = 1.97, 5.31), and suicidal ideation (OR = 2.727, 95% CI = 1.58, 4.72). The presence of very uncomfortable speaking problems was significantly associated with stress (OR = 1.592, 95% CI = 1.13, 2.24) but not significantly associated with depression and suicidal ideation.

**Conclusions:**

Oral functional problems including chewing and speaking difficulties can be associated with mental health. It is necessary to develop oral health promotion programs for adults and help them maintain a good quality of life and mental health.

## Background

Owing to the high level of economic growth and development in science and technology in our modern society, the average human life expectancy has extended, and the quality of life has improved. In the meantime, increased attention has been drawn to oral health, which is known to play an important role in promoting good mental health [1]. One interpretation of these findings is that oral health is an important contributor to the well-being of older adults, along with general health status and life circumstances.

Oral health is defined as “a state of being free of mouth and facial pain, oral and throat cancer, oral infection and sores, birth defects such as cleft lip and palate, periodontal (gum) disease, tooth decay and tooth loss, and other diseases and disorders that limit an individual’s capacity in biting, chewing, smiling, speaking, and psychosocial well-being” [2]. At a biological level, the oral cavity contributes to the quality of life by protecting the body from systemic infections and through chewing and swallowing. At the social and psychological levels, the oral cavity contributes to self-esteem, self-expression, communication, and facial aesthetics [3].

Oral health greatly affects well-being throughout the different stages of life from childhood to late adulthood [4]. Loss of teeth due to poor oral health hinders mastication, leading to poor nutrition absorption, and affects pronunciation and aesthetics, leading to interpersonal difficulties. As social activities become limited, a sense of isolation and loneliness, stress, and depression increases while happiness decreases. Consequently, the quality of life may become compromised [5–7]. Chewing and speaking, which are the subjective components of oral health, are the essential aspects of well-being. Discomfort in chewing and speaking not only limits oral functions but also greatly affects mental health [1, 6, 7].

Korean adults suffer from various mental problems due to stress from all the tasks they must complete during different stages of life. During adulthood, they experience especially high levels of stress as they must find employment and establish a family. After retirement, they are met with a sense of helplessness, loneliness, and, eventually, depression, depending on their financial status due to the loss of their role in the society. After sacrificing their lives for their families and work during their early years without investing in leisure and preparing for old age, they experience a feeling of loneliness, idleness, and depression as well as suicidal ideation once they reach old age [8–10]. The suicide rates in Korea have been 31.7, 28.1, 28.5, and 27.3 per 100,000 persons in 2011, 2012, 2013, and 2014, respectively [11].

Korea’s suicide rate is much higher than those of Turkey, Greece, Mexico, Italy, and Israel, at seven or fewer per 100,000 population, as well as those of Japan, Hungary, and Slovenia, with a high suicide rate of 20 deaths per 100,000 population [12]. Accordingly, an investigation into the causes of suicide from various angles as well as the establishment of suicide countermeasures at a national level is urgently needed.

Prevention of mental illnesses is one of the WHO’s main tasks [13], and numerous studies are looking into various factors that affect mental health. These include research on the association between oral health and mental health. Several studies have investigated this association using large data obtained from the Korea National Health and Nutrition Examination Survey (KNHANES) [5, 14–16]. A study has reported on the effects of oral health behavior and status on the quality of life in the Korean elderly [5], and only a few reports have indicated that oral pain or oral health status is correlated with depression in Korean adults or the elderly [14–16]. However, there is a lack of research on the influence of oral health status on stress, depression, and suicidal ideation-the primary markers of mental health-among Korean adults.

This study aimed to investigate the extent to which subjective oral health status and chewing/speaking difficulties are associated with the level of stress, depression, and suicidal ideation in Korean adults based on the Fifth KNHANES conducted between 2010 and 2012, and thereby gather data that may be useful for mental health management through oral health care.

## Methods

### Study population

The participants in this study were from the Fifth KNHANES, which was conducted in 2010, 2011, and 2012 by the Korea Centers for Disease Control and Prevention (KCDCP). The goal of the survey was to gather national data on the health status, health awareness and behavior, and nutritional intake status of South Korean citizens. The sampling method of the Fifth KNHANES involved a complex, stratified, multistage, probability-cluster survey of representative samples. The target population of this survey included all non-institutionalized civilian South Korean individuals [17]. The Fifth KNHANES included highly structured health-related questionnaires, a nutrition survey, and an oral health examination conducted by trained interviewers and dentists [18].

The target population of the Fifth KNHANES (2010-2012) was the total residents of Republic of Korea. Based on the 2005 Population and Housing Census conducted by Statistics Korea, 11,400 households and 31,596 members of households older than 1 year were selected from 600 geographical units. The dataset comprised 3 parts: a physical examination, a health questionnaire, and a nutritional examination. Response rates of physical examination and health questionnaire among the total target population were 77.5% in 2010, 76.1% in 2011, and 75.9% in 2012. The number of participants who completed the Fifth KNHANES was 31,596 [19]. The number of participants aged 35 years and more included in this study from among the participants of the Fifth KNHANES was 15,716. However, the final number of analyzed participants who were examined for oral health status and who answered the all questionnaires on socioeconomic status and oral health behavior was 11,347.

### Subjective oral health status

The demographic and socioeconomic variables in the survey included gender, age, household income, and education. The health behavior variables included smoking and drinking. The variables of subjective oral health status included self-perceived oral health, including teeth and gum health. The oral functional status variables were chewing and speaking, and the mental health variables were self-perceived levels of stress, depression, and suicidal ideation.

Regarding the demographic and socioeconomic variables, gender was categorized as males and females, and age was categorized as 35–44, 45–54, 55–65, 65–74, and 75 years old or more. Household income was divided into four quartiles (low, middle-low, middle-high, high) for different age/gender groups according to the mean monthly equalized household income. The level of education was divided into completion of primary school, middle school, high school, and college.

“Smoking” as a health behavior variable referred to the current smoking status. Participants who were currently smoking were classified as “smoking” while those who no longer smoked or had never smoked were classified as “non-smoking.” For the variable “drinking,” the participants were classified based on their scores on the Alcohol Use Disorders Identification Test (AUDIT) [20] that consists of 10 questions. Accordingly, based on the drinking status, participants were classified as “unhazardous” for scores 0–9, “hazardous” for 10–19, and “alcohol use disorder” for 20–40 [21].

For classification of self-perceived oral health status variables, one of the following responses to the question, “How do you feel about your oral health related to teeth and gums, etc.?” was used directly: “very good,” “good,” “fair,” “poor,” and “very poor.” For classification of the self-perceived oral functional status variables, one of the following responses to the question, “Do you experience any difficulty or discomfort when pronouncing words clearly due to problems with teeth, dentures, or gums?” was used directly: “very uncomfortable,” “uncomfortable,” “fair,” “comfortable,” and “very comfortable.”

For classification of the mental health variables, one of the following responses to the question, “How stressed are you on a daily basis?” was used directly: “extremely stressed,” “quite stressed,” “a little bit stressed,” and “not stressed at all.” For classification of the variables of self-perceived level of depression and suicidal ideation, either of the following responses to the questions, “Have you experienced a continuous feeling of sadness or despair for over 2 weeks that interfered with your daily activities in the last year?” and “Have you considered committing suicide in the last year?” was used directly: “yes” or “no.”

For the purpose of this study, mental health variables such as stress, depression, and suicidal ideation were set as the dependent variables, and the demographic and socioeconomic, health behavior, and subjective oral health status and oral functional status variables were set as the independent variables.

### Statistical analysis

In this study, data obtained from the Fifth KNHANES (2010–2012) were analyzed using a complex sample design that takes into account the characteristics of the raw data. The weighted value used in the existing health survey of the 3-year period were combined into a single value, and a plan file was created.

Stress, depression, and suicidal ideation according to the demographic and socioeconomic, health behavior, and subjective oral health and oral functional status variables were calculated with frequency (weighted and not weighted) and percentage (estimated). Differences in these values were analyzed using a chi-square test. In addition, to find the degree of influence of the demographic and socioeconomic status, health behavior, and subjective oral health and functional status variables on stress, depression, and suicidal ideation, a multiple logistic regression analysis was performed with the following variables: gender, age, household income, education, smoking, drinking, subjective oral health status, and oral functional status (chewing and speaking). The level of significance was set at *P* < 0.05.

## Results

Of the examined participants, 25.4% experienced stress, and the rate of stress was high among female participants who were aged between 35 and 44 years, had completed college, were smokers, or had an alcohol use disorder. Thirteen percent of the participants experienced depression, and the rate of depression was high among female participants who were aged 75 years or more, were in the fourth quartile of household income, had completed primary school, or had an alcohol use disorder. The rate of depression was not significantly associated with smoking status. Finally, 13.9% of the participants experienced suicidal ideation, and the level of suicidal ideation was high among female participants who were aged 75 years or more, were in the fourth quartile of household income, had completed primary school, and had an alcohol use disorder. The level of suicidal ideation was not significantly related to smoking status (Table 1).

**Table 1:** Stress, depression, and suicidal ideation by demographic and socioeconomic status and health behavior

Parameters	Categories	*N*	Stress (%)^a^		*P* ^b^	Depression (%)^a^		*P* ^b^	Suicidal ideation (%)^a^		*P* ^b^
			Inexperienced^a^	Experienced^a^		Inexperienced^a^	Experienced^a^		Inexperienced^a^	Experienced^a^	
Total		11,347	74.6	25.4		87.0	13.0		86.1	13.9	
Gender	Female	5,838	72.4	27.6	<0.001	83.4	16.6	<0.001	82.7	17.3	<0.001
	Male	5,509	76.6	23.4		90.3	9.7		89.0	11.0	
Age group	35–44	3,227	70.8	29.2	<0.001	90.5	9.5	<0.001	90.6	9.4	<0.001
	45–54	2,773	73.3	26.7		85.5	14.5		85.7	14.3	
	55–64	2,504	79.6	20.4		84.7	15.3		84.9	15.1	
	65–74	1,994	79.4	20.6		86.3	13.7		81.0	19.0	
	75≤	849	79.9	20.1		84.6	15.4		73.9	26.1	
Household	Low	2,243	72.3	27.7	0.092	79.9	20.1	<0.001	75.2	24.8	<0.001
income^c^	Low-middle	2,882	75.9	24.1		86.6	13.4		85.3	14.7	
	Middle-high	3,037	75.5	24.5		89.8	10.2		89.2	10.8	
	High	3,185	73.9	26.1		89.0	11.0		90.1	9.9	
Education	Primary	3,068	74.5	25.5	<0.001	81.5	18.5	<0.001	76.2	23.8	<0.001
	Middle	1,457	79.6	20.4		84.9	15.1		85.2	14.8	
	High	3,737	75.6	24.4		88.4	11.6		88.8	11.2	
	College≤	3,085	71.2	28.8		90.8	9.2		90.9	9.1	
Smoking	Smoking	2,462	70.3	29.7	<0.001	86.6	13.4	0.533	85.2	14.8	0.212
	Non-smoking	8,885	76.2	23.8		87.2	12.8		86.4	13.6	
Drinking	Unhazardous (0–9)	8,583	75.9	24.1	<0.001	87.5	12.5	0.006	86.2	13.8	<0.001
AUDIT^d^(0-40)	Hazardous (10–19)	2,119	73.5	26.5		87.4	12.6		87.7	12.3	
	Disorder (20–40)	645	65.5	34.5		81.9	18.1		79.8	20.2	

^a^Estimate %

^b^Complex Samples chi-square test

^c^Gross household income divided by the square root of the number of household members

^d^Alcohol use disorder identification

The rates of stress, depression, and suicidal ideation were highest for those with “very poor” self-perceived oral health status and “very uncomfortable” oral functions (chewing and speaking) (Table 2).

**Table 2:** Stress, depression, and suicidal ideation by subjective oral health status and oral functions

Parameters	Categories	*N*	Stress (%)^a^		*P* ^b^	Depression (%)^a^		*P* ^b^	Suicidal ideation (%)^a^		*P* ^b^
			Inexperienced^a^	Experienced^a^		Inexperienced^a^	Experienced^a^		Inexperienced^a^	Experienced^a^	
Total		11,347	74.6	25.4		87.0	13.0		86.1	13.9	
Oral health	Very poor	1,066	70.8	29.2	<0.001	81.6	18.4	<0.001	81.0	19.0	<0.001
perception	Poor	4,394	71.0	29.0		85.4	14.6		84.3	15.7	
	Fair	4,373	78.1	21.9		88.8	11.2		87.7	12.3	
	Good	1,367	78.1	21.9		91.5	8.5		90.1	9.9	
	Very good	147	78.6	21.4		87.4	12.6		91.1	8.9	
Chewing	Very uncomfortable	546	68.5	31.5	<0.001	76.3	23.7	<0.001	72.7	27.3	<0.001
	Uncomfortable	2,762	70.7	29.3		84.3	15.7		81.8	18.2	
	Fair	1,758	72.7	27.3		85.8	14.2		84.5	15.5	
	Comfortable	2,985	75.8	24.2		87.7	12.3		88.1	11.9	
	Very comfortable	3,296	78.5	21.5		90.8	9.2		90.3	9.7	
Speaking	Very uncomfortable	135	56.9	43.1	<0.001	61.9	38.1	<0.001	58.4	41.6	<0.001
	Uncomfortable	893	72.3	27.7		81.7	18.3		77.4	22.6	
	Fair	945	69.6	30.4		79.0	21.0		77.9	22.1	
	Comfortable	3,363	75.8	24.2		86.4	13.6		85.4	14.6	
	Very comfortable	6,011	75.4	24.6		89.7	10.3		89.2	10.8	

^a^Estimate %

^b^Complex Samples chi-square test

The rate of stress was 1.777 times higher among females than male participants and was reduced by 0.975 times after each year. Regarding household income, the rate of stress was 1.261 times higher in the lowest quartile than in the highest quartile. Regarding the education level, the rates were 0.672 and 0.766 times lower among participants who had completed middle school and high school education, respectively, than among those who had completed college. The rate of stress was also 1.394 times higher among smokers than non-smokers, and it increased by 1.027 times with every point gained on the AUDIT. While the rate of stress was related to subjective oral health status, it was 2.294 times higher for “very uncomfortable” chewing compared to “very comfortable” chewing and 1.592 times higher for “very uncomfortable” speaking compared to “very comfortable” speaking.

The rate of depression was 2.849 times higher among female participants than in male participants, 1.513 times higher in the lowest household income quartile than in the highest household income quartile, and 1.372 and 1.332 times higher among primary and middle school graduates, respectively, than among college graduates. The rate of depression was 1.346 higher among smokers than among non-smokers, and it increased by 1.045 times with every point gained on the AUDIT. In addition, the rates of depression were 3.232, 1.431, 1.773, and 1.217 times higher for “very uncomfortable,” “uncomfortable,” “fair,” and “comfortable” chewing, respectively, compared to “very comfortable” chewing. However, age, subjective oral health status, and speaking were not associated with depression.

The rate of suicidal ideation was 2.460 times higher among females than among male participants, and it increased by 1.009 times in each year. The rates of suicidal ideation were 1.859 and 1.297 times higher in the low and middle-low household income quartiles, respectively than in the high quartile; it was 1.535 times higher for completion of primary school than for completion of college. It was 1.457 times higher among smokers than non-smokers and increased by 1.039 times with every point gained on the AUDIT. The rates of suicidal ideation were 2.727, 1.499, 1.612, and 1.232 times higher for “very uncomfortable,” “uncomfortable,” “normal,” and “comfortable” chewing, respectively, compared to “very comfortable” chewing. Subjective oral health status and speaking did not affect suicidal ideation (Table 3).

**Table 3:** Predictors of stress, depression, and suicidal ideation among Korean adults

Parameters	Categories	Stress^a^			Depression^a^			Suicidal Ideation^a^		
		OR^b^	95% CI^c^	*P* ^d^	OR^b^	95% CI^c^	*P* ^d^	OR^b^	95% CI^c^	*P* ^d^
(Intercept)		0.605	0.33–1.11	0.107	0.047	0.02–0.09	<0.001	0.019	0.01–0.05	<0.001
Gender	Female	1.777	1.55–2.03	<0.001	2.849	2.33–3.48	<0.001	2.460	2.05–2.96	<0.001
	Male (reference)	1.000			1.000			1.000		
Age		0.975	0.97–0.98	<0.001	0.999	0.99–1.01	0.782	1.009	1.00–1.02	0.037
Household income^e^	Low	1.261	1.05–1.51	0.013	1.513	1.19–1.93	0.001	1.859	1.47–2.34	<0.001
	Low-middle	0.892	0.77–1.04	0.137	1.055	0.84–1.32	0.640	1.297	1.04–1.61	0.020
	Middle-high	0.898	0.77–1.04	0.163	0.875	0.71–1.08	0.220	1.051	0.86–1.29	0.632
	High (reference)	1.000			1.000			1.000		
Education	Primary	0.960	0.80–1.15	0.654	1.372	1.06–1.77	0.016	1.535	1.19–1.98	0.001
	Middle	0.672	0.55–0.82	<0.001	1.332	1.04–1.71	0.024	1.149	0.88–1.49	0.297
	High	0.766	0.67–0.88	<0.001	1.070	0.87–1.32	0.523	0.989	0.79–1.24	0.921
	≥College (reference)	1.000			1.000			1.000		
Smoking	Smoking	1.394	1.20–1.62	<0.001	1.346	1.10–1.65	0.004	1.457	1.20–1.77	<0.001
	Non-smoking (reference)	1.000			1.000			1.000		
Drinking AUDIT^f^ (0–40)		1.027	1.02–1.04	<0.001	1.045	1.03–1.06	<0.001	1.039	1.03–1.05	<0.001
Oral health perception	Very poor	1.114	0.63–1.96	0.707	0.971	0.53–1.78	0.925	1.366	0.67–2.80	0.393
	Poor	1.230	0.71–2.12	0.454	0.878	0.50–1.55	0.653	1.321	0.66–2.63	0.427
	Fair	0.906	0.53–1.54	0.716	0.748	0.43–1.31	0.312	1.258	0.62–2.56	0.525
	Good	1.061	0.60–1.86	0.838	0.617	0.34–1.13	0.117	1.037	0.50–2.16	0.924
	Very good (reference)	1.000			1.000			1.000		
Chewing	Very uncomfortable	2.294	1.41–3.72	0.001	3.232	1.97–5.31	<0.001	2.727	1.58–4.72	<0.001
	Uncomfortable	1.131	0.91–1.40	0.265	1.431	1.09–1.89	0.011	1.499	1.16–1.94	0.002
	Fair	1.202	0.98–1.48	0.083	1.773	1.37–2.30	<0.001	1.612	1.26–2.06	<0.001
	Comfortable	0.897	0.77–1.04	0.156	1.217	1.01–1.47	0.040	1.232	1.01–1.50	0.036
	Very comfortable (reference)	1.000			1.000			1.000		
Speaking	Very uncomfortable	1.592	1.13–2.24	0.008	1.329	0.92–1.93	0.134	1.340	0.92–1.95	0.126
	Uncomfortable	1.568	1.31–1.88	<0.001	1.196	0.96–1.49	0.113	1.245	0.98–1.58	0.075
	Fair	1.393	1.14–1.70	0.001	1.116	0.87–1.43	0.384	1.132	0.87–1.47	0.357
	Comfortable	1.277	1.07–1.52	0.007	1.108	0.88–1.40	0.391	0.957	0.74–1.25	0.746
	Very comfortable (reference)	1.000			1.000			1.000		

Model: (Intercept), gender, age, household income, education, smoking, drinking, oral health perception, chewing, speaking

Naqelkerke R squares = 0.062

Covariated mean: age = 51.57, AUDIT = 6.80

^a^Reference: inexperienced = 0

^b^Odds ratio

^c^95% CI = 95% Confidence Interval

^d^Using complex samples logistic regression

^e^Gross household income divided by the square root of the number of household members

^f^Alcohol use disorder identification

## Discussion

Based on the demographic and socioeconomic status, the rate of stress was higher among female participants of the younger age groups from the low quartile of household income and who had completed college education. The rate of stress found in this study was lower than that reported in a previous study (27.96%) [22], which used the Fourth KNHANES (2008) data. However, the rate reported by the previous study was pertinent to the 40–55-year-old age group only, and the difference between the rates of the two studies may be due to the different age groups studied. In this study, the rate of stress among the older participants was higher than among younger participants. Based on the trend among Koreans from 2008 to 2013, the rate of stress was high among young Koreans aged 39 years or below [23], an important stage of life during which they must go through employment, marriage, and child-rearing. In addition, the rate of stress was higher among women aged 35–44 years old than among men, and this is consistent with a report that women experience a greater degree of stress than men due to child-rearing, housekeeping, and work, along with the physical and psychological stress due to pregnancy and childbirth [24]. A social investigation report by the National Statistics Office reported higher levels of stress among those with high educational backgrounds [25], which was also observed in this study. In addition, a study on Korean office workers reported that the higher the level of one’s education, the more pressured and stressed one felt at work [26]. This finding supports the result of this study.

The rate of depression was higher among female participants who were in the low quartile of household income and had completed primary school education only. The rate of self-perceived depression found in this study was lower than that reported by a previous study (15.6%) [27] that used the Fourth KNHANES (2008) data; however, this rate is pertinent to the 40–55-year-old age group only, and, therefore, one should be careful while interpreting the results. In this study, the rate of depression was 16.6% among women, which was 2.849 higher than the rates reported in 2008 [22]. This is consistent with a study that used Goldberg’s depression scale [28] that reported rates of “helplessness” at 33.9% (males) and 43.1% (females), of “calm down” at 30.1% (males) and 37.7% (females), and of “hopelessness” at 11.7% (males) and 17.7% (females). The rate of depression was higher among women than men in both the studies [28]. A study using the Third KNHANES (2005) [22] data reported that the rate of depression was high among women in their 60s with a monthly family income of less than one million Korean won (990 USD). While this finding was similar to our study result, it is inconsistent with the result of a study that used the first and second year of Fourth KNHANES (2007–2008) data, which reported no differences in the rate of depression for different ages, household incomes, and educational backgrounds [9].

The rate of suicidal ideation was higher among female participants who were aged 75 years and over, were in the low quartile of household income, and had completed primary school education only. In a previous study that used the Second KNHANES (2001) data, the rate of suicidal ideation was 18.6% [8]; the rate was 21.4% in a study that used the Fourth KNHANES data [9] and 16.9% in a study that involved employed Koreans from 10 different regions [10]. These rates were higher than that found in this study. However, the rate of suicidal ideation of this study is similar to that reported by an Australian national research study (13.3%) [29]. In addition, in this study, the rate of suicidal ideation was higher among women than men. In an Australian study that involved local residents of an Australian community, the rate of suicidal ideation was 15.5% for men and 18.1% for women, and the rate of planning suicide was 0.7% for men and 1.0% for women [30]. One of the findings of this study that women experience suicidal ideation 2.46 times more frequently than men is consistent with the results of many previous studies [8]. Thus, a more active and thorough approach to the prevention of suicidal ideation is needed.

In health behavior, the rates of stress, depression, and suicidal ideation were higher among smokers than among non-smokers, and it was high among those with alcohol use disorders. This finding is not consistent with a previous study [9] that used the Third KNHANES data, which reported that drinking was not related to depression while smoking was. Pratt et al. [30] reported that adults aged 20 years and over with depression were more likely to be cigarette smokers than those without depression. Boden et al. [31] reported that adolescent major depression/alcohol use disorder comorbidity is likely to be a risk factor rather than a causal factor of subsequent major depression. The rate of suicidal ideation was significantly higher among participants with alcohol use disorders than among those with other alcohol-related problems or those with no problems at all. Since lifestyle factors greatly influence smoking and drinking behavior, and drinking and smoking are used as common ways to relieve stress, it is difficult to explain whether or not smoking and drinking are associated with mental health. Studies examining the reasons for smoking and drinking should attain a more in-depth understanding of the association of drinking and smoking with mental health.

Subjective oral functional status composed of chewing was shown to be associated with stress, depression, and suicidal ideation; on the other hand, speaking was shown to be only associated with stress. A study involving high school students reported that the higher the level of stress from university entrance examination competition, the higher the perception of the oral diseases [32]. Another study involving middle-aged Swedish women reported no association between oral health and level of stress [33]. Vasiliou et al. [34] reported that a positive relationship between current stress and self-reported poor oral health was observed. A study involving old Brazilian men reported that unpleasant oral conditions such as dry mouth, oral pain, and edema increased the risk of depression [35]. This is also consistent with the study on the association between the number of remaining teeth with depression among Korean adults, which indicated that the risk of depression could increase by up to 1.94 times as the number of the remaining teeth decreases [36]. Roohafza et al. [37] reported that participants with higher scores on depression, anxiety, and stress suffer from lower masticatory ability. A study that involved young Australian natives reported that poor oral health status was associated with suicide [38], and, therefore, it can be said that oral functional status as an associated factor of suicidal ideation is a rather new discovery. More research must be done to clarify the existence of this association. This suggests that variable of oral functional status including chewing and speaking have a huge impact on stress, depression, and suicidal ideation, and therefore, the promotion of oral health may be conducive to mental health improvement.

The following deductions can be made from the analysis of the association of the demographic and socioeconomic status, health behavior, and subjective oral health and functional status variables with stress, depression, and suicidal ideation. First, this study confirmed that subjective oral health status was not significantly associated with the rates of stress, depression, and suicidal ideation. However, uncomfortable chewing had a huge influence on stress, depression, and suicidal ideation. Uncomfortable speaking was greatly associated with stress but not with depression and suicidal ideation. In other words, the variables of oral functional status including chewing and speaking rather than the subjective oral health status were greatly associated with mental health in Korean adults. These results suggest that oral health education programs and preventive care should begin early during childhood, and oral health programs to prevent oral diseases and tooth loss are needed. Second, with regard to mental health, the rate of stress and depression was high among the younger age groups whereas the rate of suicidal ideation was higher among the older age groups. The rate of self-perceived stress and depression and suicidal ideation was higher among women than men. Therefore, we suggest that mental health care catering to different age groups as well as programs designed to help improve mental health in women should be developed. Since the rates of self-perceived stress, depression, and suicidal ideation were high among participants with a low household income, measures to bring up the household income of the lower class should be developed at the national level. One of the limitations of this study is the employment of only a cross-sectional design. If a time-series design is used for this study, better results with higher explanatory power can be expected.

## Conclusions

Oral functional problems including chewing and speaking difficulties can be associated with mental health. It is necessary to develop oral health promotion programs for adults and help them maintain a good quality of life and mental health.

## Abbreviations

AUDIT: Alcohol Use Disorders Identification Test
CI: Confidence Interval
KCDCP: Korea Centers for Disease Control and Prevention
KNHANES: Korea National Health and Nutrition Examination Survey
OECD: Organization for Economic Cooperation and Development
OR: Odds Ratio
WHO: World Health Organization
